# Microcalcification and ^99m^Tc-Pyrophosphate Uptake without Increased Bone Metabolism in Cardiac Tissue from Patients with Transthyretin Cardiac Amyloidosis

**DOI:** 10.3390/ijms24031921

**Published:** 2023-01-18

**Authors:** Atsushi Mori, Yukihiro Saito, Kazufumi Nakamura, Toshihiro Iida, Satoshi Akagi, Masashi Yoshida, Makiko Taniyama, Toru Miyoshi, Hiroshi Ito

**Affiliations:** 1 Department of Cardiovascular Medicine, Graduate School of Medicine, Dentistry and Pharmaceutical Sciences, Okayama University, Okayama 700-8558, Japan; 2 Department of Cardiovascular Medicine, Okayama University Hospital, Okayama 700-8558, Japan; 3 Department of Cardiovascular Medicine, Faculty of Medicine, Dentistry and Pharmaceutical Sciences, Okayama University, Okayama 700-8558, Japan; 4 Department of Chronic Kidney Disease and Cardiovascular Disease, Faculty of Medicine, Dentistry and Pharmaceutical Sciences, Okayama University, Okayama 700-8558, Japan; 5 Department of General Medicine, Tamano Division, Faculty of Medicine, Dentistry and Pharmaceutical Sciences, Okayama University, Okayama 700-8558, Japan

**Keywords:** ATTR, ^99m^Tc-labeled bone scintigraphy, calcified microparticle

## Abstract

Transthyretin cardiac amyloidosis (ATTR-CA) is characterized by high ^99m^Tc-labeled bone tracer uptake in the heart. However, the mechanism of bone tracer uptake into the heart remains controversial. Since bone tracer uptake into metastatic bone tumors is thought to be associated with increased bone metabolism, we examined ^99m^Tc-pyrophosphate (PYP) scintigraphy findings, endomyocardial biopsy (EMB) tissue findings, and the expression of bone metabolism-related genes in the EMB tissues in patients with ATTR-CA, amyloid light-chain cardiac amyloidosis (AL-CA), and noncardiac amyloidosis (non-CA) in this study. The uptake of ^99m^Tc-PYP in the heart was significantly higher in the ATTR-CA patients than in the AL-CA and non-CA patients. A higher percentage of ATTR-CA EMB tissue showed von Kossa-positive microparticles: ATTR-CA, 62%; AL-CA, 33%; and non-CA, 0%. Calcified microparticles were identified using transmission electron microscopy. However, none of the osteogenic marker genes, osteoclastic marker genes, or phosphate/pyrophosphate-related genes were upregulated in the EMB samples from ATTR-CA patients compared to those from AL-CA and non-CA patients. These results suggest that active calcification-promoting mechanisms are not involved in the microcalcification observed in the heart in ATTR-CA. The mechanisms explaining bone tracer uptake in the heart, which is stronger than that in the ribs, require further investigation.

## 1. Introduction

Cardiac amyloidosis is a disease in which amyloid, a water-insoluble fibrous protein rich in β-sheet structures, is deposited in the heart, resulting in symptoms of heart failure [1]. The main types of cardiac amyloidosis include transthyretin cardiac amyloidosis (ATTR-CA), in which transthyretin (TTR) is the amyloid precursor, and amyloid light-chain cardiac amyloidosis (AL-CA), in which immunoglobulin light chains are the amyloid precursor [2,3]. TTR circulates in the blood as a stable tetramer; however, monomers or oligomers generated through aging or *TTR* gene mutations form amyloid fibrils and cause organ damage [4]. With advances in diagnostic and therapeutic methods [5,6,7,8], early diagnosis of ATTR-CA is becoming increasingly important. Scintigraphy with ^99m^Tc-labeled bone tracers is particularly useful for the diagnosis of ATTR-CA [9,10]. In addition, it may be useful in predicting the disease prognosis [11,12]. Elucidating the mechanism of bone tracer uptake will not only make the test more useful for diagnosis but may also clarify the pathophysiology and lead to new treatments. However, it is known that cardiac uptake of bone tracer is poor in patients with certain mutations of the *TTR* gene [13], and the mechanism of bone tracer uptake is still unclear.

Bone scintigraphy has been used to diagnose diseases involving active bone metabolism and inflammation (osteogenic metastatic bone tumors, fractures, and inflammatory bone and joint diseases) [14]. Interestingly, microcalcifications have been reported in the cardiac tissues of patients with ATTR-CA, suggesting that this calcification may be the cause of bone tracer uptake [15,16]. However, some studies have suggested that calcification alone does not fully explain the cardiac uptake of bone tracers and may reflect differences in the amount or type of amyloid fibrils [3,17,18]. Alternatively, high levels of inflammatory cytokines in the blood of patients with familial amyloid neuropathy [19] and increased expression of inflammatory cytokines when cardiac fibroblasts are cultured with TTR amyloid fibers synthesized in vitro [20] suggest that inflammation may be involved in the pathogenesis of ATTR-CA. In addition, ectopic calcification associated with inflammation is accompanied by increased expression of bone metabolism-related genes [21].

Therefore, in this study, we investigated the mechanisms of bone tracer uptake and microcalcification in the hearts of patients with ATTR-CA, both histologically and molecularly.

## 2. Results

### 2.1. Cardiac Uptake of ^99m^Tc-Pyrophosphate

**Figure 1:**
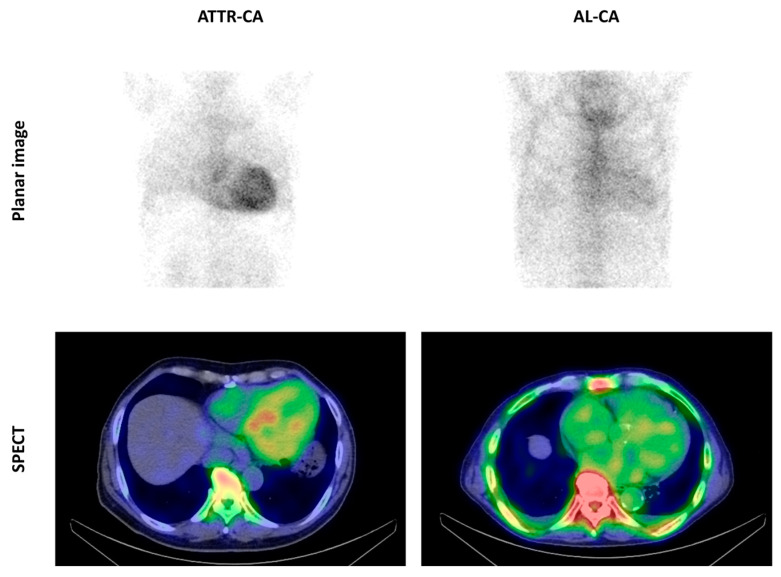
Cardiac uptake of ^99m^Tc-PYP in patients with cardiac amyloidosis. The upper left shows a planar image of a patient with transthyretin cardiac amyloidosis (ATTR-CA) and the lower left shows a SPECT image of the same patient. The upper right shows a planar image of a patient with amyloid light-chain cardiac amyloidosis (AL-CA), and the lower right shows a SPECT image of the same patient. The heart-to-contralateral ratios were 2.0 in the patient with ATTR-CA and 1.2 in the patient with AL-CA.

Figure 1 shows representative images of the cardiac uptake of ^99m^Tc-pyrophosphate (^99m^Tc-PYP) in patients with ATTR-CA and AL-CA. Patients with ATTR-CA showed substantially higher cardiac uptake of ^99m^Tc-PYP than AL-CA patients and noncardiac amyloidosis (non-CA) patients (Table 1).

### 2.2. Microcalcification Detected by Von Kossa Staining

**Figure 2:**
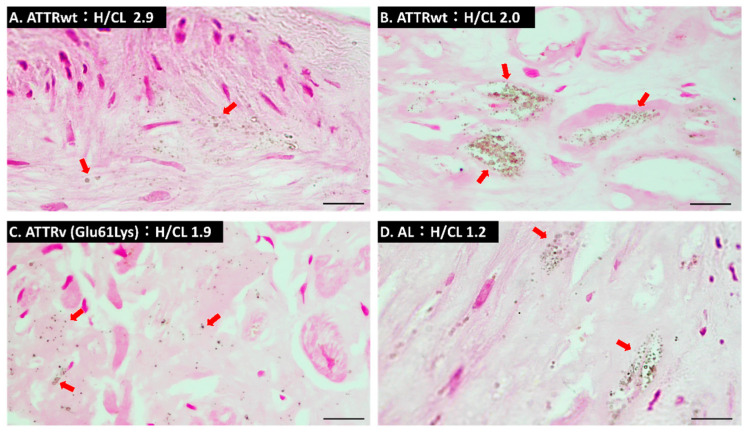
von Kossa staining of myocardial tissues. (**A**) von Kossa staining of a slice obtained from a patient with wild-type transthyretin cardiac amyloidosis (ATTRwt-CA) whose heart-to-contralateral (H/CL) ratio was 2.9. (**B**) von Kossa staining of a slice obtained from a patient with ATTRwt-CA whose H/CL ratio was 2.0. (**C**) von Kossa staining of a slice obtained from hereditary ATTR (ATTRv-CA) whose H/CL ratio was 1.9. (**D**) von Kossa staining of a slice obtained from a patient with amyloid light-chain cardiac amyloidosis (AL-CA) whose H/CL ratio was 1.2. Red arrows indicate von Kossa-positive microparticles. Scale bars are 10 µm.

As shown in Table 2, compared to AL-CA and non-CA, a higher percentage of ATTR-CA endomyocardial biopsy tissue showed von Kossa stain-positive microparticles, but there was not a statistically significant difference between patients with ATTR-CA and AL-CA (ATTR-CA, 8/13 [62%]; AL-CA, 2/6 [33%]; Non-CA, 0/6 [0%]). In most samples, von Kossa-positive microparticles were observed more frequently in fibrotic areas than in areas with residual myocardial tissue (Figure 2A,B). In addition, in the sample of a patient with hereditary ATTR-CA with the Glu61Lys TTR mutation, calcified particles were also observed in the interstitial area surrounding cardiomyocytes (Figure 2C). In a sample of an AL-CA patient with heart-to-contralateral (H/CL) ratio as low as 1.2, von Kossa-positive microparticles were observed (Figure 2D).

There were no differences in renal function or serum calcium levels related to ectopic calcification among the three groups (Table 3).

### 2.3. Findings of Transmission Electron Microscopy

**Figure 3:**
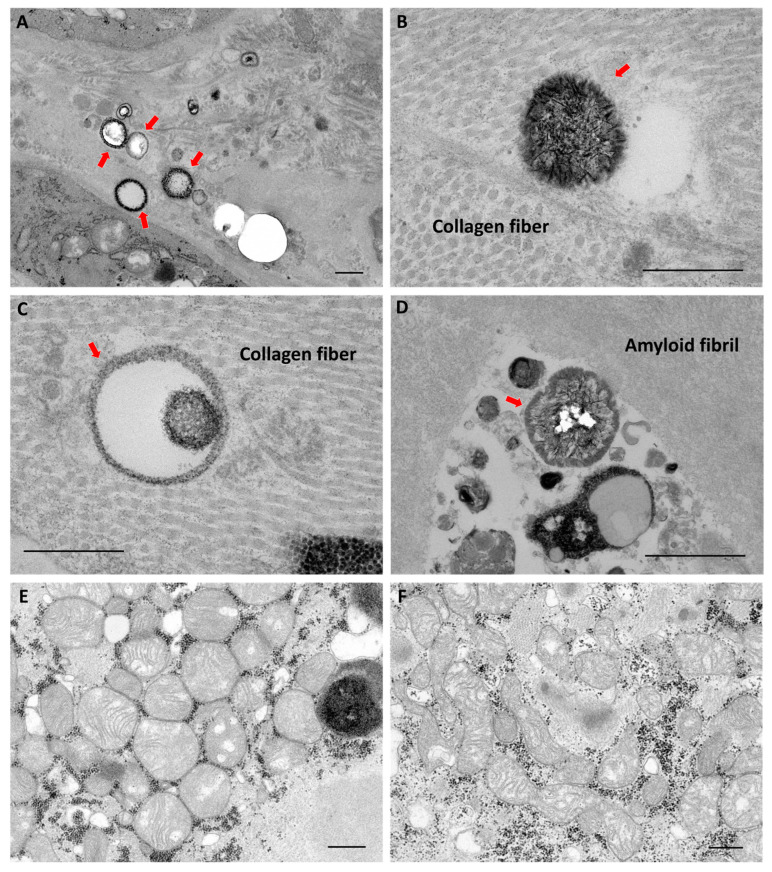
Transmission electron microscopy findings using the myocardial tissue from a patient with transthyretin cardiac amyloidosis. (**A**–**D**) Red arrows indicate calcified microparticles. (**E**,**F**) Calcification of mitochondria in cardiomyocytes were not observed. The black granules surrounding the mitochondria are glycogen granules. Scale bars are 500 nm.

Two ATTR-CA, two AL-CA, and two non-CA samples were examined, and calcified microparticles were only observed in one ATTR-CA sample using transmission electron microscopy (TEM). As shown in Figure 3A–D, calcified particles were present at sites surrounded by collagen fibers and at the boundaries or gaps between collagen and amyloid fibers. In contrast, no particles were clearly present in amyloid fibers or in the mitochondria of cardiomyocytes (Figure 3E,F).

### 2.4. Bone Metabolism-Related Gene Expression

**Figure 4:**
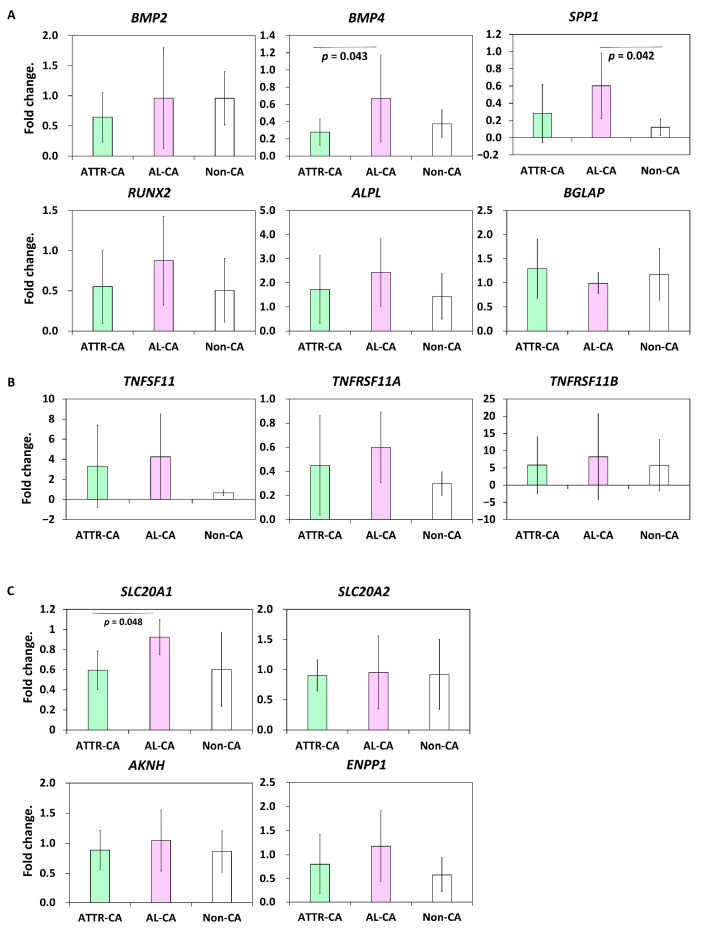
Comparison of bone metabolism-related gene expressions in endomyocardial biopsy samples. (**A**) Expression levels of osteogenic markers. (**B**) Expression levels of osteoclastic gene markers. (**C**) Expression levels of phosphate and pyrophosphate-related genes. N = 11 in the transthyretin cardiac amyloidosis (ATTR-CA) group, N = 6 in the amyloid light-chain cardiac amyloidosis (AL-CA) group, and N = 6 in the noncardiac amyloidosis (non-CA) group. ANOVA/Bonferroni test was performed.

Although calcium deposition was indeed observed in the cardiac tissue of patients with ATTR-CA, higher accumulation of bone tracer was observed in the heart than in the ribs, which are calcium clumps [9]. Bone scintigraphy is useful for the diagnosis of bone metastases of malignant tumors because bone metabolism and osteogenesis are enhanced at the sites of bone tracer accumulation. Therefore, we investigated the expression of bone metabolism-related genes in cardiac tissues. However, the gene expressions of osteogenic markers (*BMP2*, *BMP4*, *RUNX2*, *ALPL*, *SPP1*, and *BGLAP*), osteoclastic markers (*TNFSF11*, *TNFRSF11A*, and *TNFRSF11B*), or phosphate and pyrophosphate-related genes (*SLA20A1*, *SLA20A2*, *AKNH*, and *ENPP1*) were not upregulated in cardiac tissues from patients with ATTR-CA compared to those from AL-CA and non-CA patients (Figure 4). On the other hand, *SPP1*, which encodes osteopontin, a protein involved in chronic inflammation and bone metabolism [22], was significantly upregulated only in cardiac tissues from patients with AL-CA (Figure 4A).

## 3. Discussion

In the present study, a higher uptake of ^99m^Tc-PYP in the heart was observed in patients with ATTR-CA than in AL-CA and non-CA patients; a higher frequency of von Kossa-positive microparticles was observed in endomyocardial biopsy samples from patients with ATTR-CA, and calcified particles were also observed using electron microscopy. Within the electron microscopic range, these calcified particles were found in collagen fiber sites but not in amyloid fibers. These findings were consistent with those reported by Stats et al. and Thelander et al. [15,16]. Pyrophosphate has a high affinity for calcium; in a canine model of myocardial infarction, calcification of mitochondria in cardiomyocytes occurred in the acute phase, and a correlation between ^99m^Tc-PYP uptake and mitochondrial calcification has been reported [23]. In contrast, mitochondrial calcification was not observed in the cardiac tissues of patients with ATTR-CA. Taken together, the cardiac uptake of ^99m^Tc-PYP in patients with ATTR-CA seems to reflect the deposition of calcified microparticles. On the other hand, ATTR-CA detection using ^18^F-sodium fluoride positron emission tomography, which detects active calcification, has been investigated, but compared to scintigraphy using ^99m^Tc-labeled bone tracers, uptake in the myocardium is much lower than that in the ribs [24,25]. Thus, it is unlikely that calcified microparticles alone are sufficient to explain why ^99m^Tc-labeled bone tracers are taken up by the heart rather than into the ribs, which have a higher total calcium content [17].

Thelander et al. speculated that the calcified microparticles observed in ATTR-CA are membrane-embedded vesicles, suggesting that the microparticles are released from within cells [16]. Bone scintigraphy is also useful in the diagnosis of metastatic bone tumors and fractures, and the uptake of bone tracers at these sites is thought to visualize increased bone metabolic turnover and bone remodeling [26]. Based on these findings, we hypothesized that calcium is not deposited passively into the heart of patients with ATTR-CA but is actively deposited by enhanced osteogenic metabolism in heart cells. However, in this study, we did not find increased expressions of genes involved in bone metabolism in the cardiac tissues of patients with ATTR-CA. Furthermore, the expression of *SPP1*, which is involved in chronic inflammation, was significantly elevated only in cardiac tissues from patients with AL-CA in this study. Since higher levels of macrophage infiltration into cardiac tissue [15] and higher T2-weighted magnetic resonance imaging signal intensity suggesting myocardia edema [27] have been reported in patients with AL-CA than in those with ATTR-CA, the degree of chronic inflammation in the heart should be stronger in patients with AL-CA than in those with ATTR-CA. These findings suggest that the microcalcification in the heart of patients with ATTR-CA is due to passive calcium deposition rather than an active calcification process associated with chronic inflammation. Unfortunately, we were unable to determine the mechanism of why there is a higher bone tracer uptake in the heart rather than in the bone of patients with ATTR-CA. On the other hand, it remains possible that differences in calcium chemistry and surface area could explain differences in affinity for tracers.

It remains unclear where bone tracers are actually taken up by the cardiac tissue. ^99m^Tc-PYP has been reported to be taken up in areas of ischemia-reperfusion injury after brief ischemia. This uptake of ^99m^Tc-PYP may be associated with increased Ca^2+^ concentration in damaged myocardial cells [28,29]. Therefore, the possibility of tracer uptake in cardiomyocytes damaged by degenerated TTR and amyloid fibrils also needs to be investigated [30]. Thelander et al. performed autoradiography using ^99m^Tc-dicarboxypropane diphosphonate (DPD) on fixed cardiac tissue sections from patients with ATTR-CA and found a tracer distribution similar to that observed with von Kossa stain [16]. However, the possibility of tracer uptake into cardiomyocytes cannot be ruled out because this study was conducted on fixed samples.

There are two types of amyloid fibrils. Type A fibrils are composed of a mixture of N-terminal truncated and full-length TTR; type B fibers are composed primarily of full-length TTR [31,32]. Type A fibrils are more common and are found in wild-type ATTR (ATTRwt) and most hereditary ATTR (ATTRv). Type B fibrils, on the other hand, form from mutant forms of Val30Met and Tyr114Cys TTRs and can produce either type A or type B fibrils, depending on the patient. Pilebro et al. reported a high rate of accumulation of ^99m^Tc-DPD in the hearts of patients with ATTR-CA with type A fibrils but not in the hearts of patients with ATTR-CA with type B fibrils. They also reported no accumulation in the hearts of ATTR-CA patients with type A fibrils [18]. In addition, ATTR-CA has been reported to deposit more amyloid fibrils than AL-CA [3], and differences in the deposition of amyloid fibers may be involved in the uptake of ^99m^Tc-labeled bone tracers. In an experiment with rats by George et al., ^99m^Tc-PYP scintigraphy was performed 1 h after the administration of aggregated TTR to the heart, and the decrease in deposition by the anti-aggregated TTR antibody could be evaluated [33]. Moreover, it has been reported that TTR binds to Ca^2+^ under high calcium concentrations and aggregates in vitro [34,35]. These findings suggest that ^99m^Tc-labeled bone tracers may bind and accumulate to amyloid itself or to Ca^2+^ bound to the amyloid. Interestingly, chelation of macromolecules by technetium has been reported to be involved in tracer uptake into the infarcted lesion [36]. This suggests that ^99m^Tc may bind to amyloids.

Limitations of this study include the following: (1) the myocardial biopsy sample was a very small tissue and might not fully reflect the characteristics of the disease, and (2) the degree of amyloid deposition and the frequency of calcified microparticles in the right ventricle, from which biopsy was obtained, might differ from that in the left ventricle [37]. On the other hand, although the number of samples in each group was small, the results were similar to those of previous reports, and it seems to corroborate the notion that calcified microparticles are deposited more frequently in ATTR-CA. In addition, none of the ATTR-CA patients we diagnosed had Val30Met or Phe64Leu TTR mutation. It has been reported that some patients with these mutations have very poor tracer uptake in ^99m^Tc-DPD or ^99m^Tc-hydroxy methylene diphosphonate scintigraphy [12,18], and a better understanding of the cardiac uptake of ^99m^Tc-labeled bone tracers and formation of calcified microparticles would be enhanced if the presence of calcified microparticles in the heart of these patient groups could be studied.

## 4. Materials and Methods

### 4.1. Patients

Japanese patients admitted to the Okayama University Hospital for the identification of cardiac amyloidosis were included in this study. Of the 49 patients who required a differential diagnosis of cardiac amyloidosis between August 2009 and August 2022, we compared pyrophosphate scintigraphy findings in 43 patients, myocardial biopsy tissue findings in 31 patients (von Kossa staining in 25 patients and TEM in 6 patients), and bone metabolism-related gene expressions in 23 patients. The ATTR-CA group included one case of ATTRv with a Glu61Lys TTR mutation, and the others were ATTRwt. Details are shown in Supplementary Table S1.

### 4.2. ^99m^Tc-Pyrophosphate Scintigraphy

Bone scintigraphy using ^99m^Tc-PYP was performed. Visual grade scoring was evaluated according to Perugini’s method: grade 0, no cardiac uptake and normal bone uptake; grade1, cardiac uptake, lower than bone uptake; grade 2 cardiac uptake associated with reduced bone uptake; or grade 3, strong cardiac uptake with mild or absent bone uptake [9]. The H/CL ratios on ^99m^Tc-PYP planar images were calculated for quantitative evaluation.

### 4.3. Right Ventricular Endomyocardial Biopsy

Right heart catheterization and an endomyocardial biopsy were performed. Biopsies were obtained from the right ventricular septum.

### 4.4. Von Kossa Staining

Five micrometer-thick sections were prepared from the paraffin-embedded myocardial biopsy samples from 13 ATTR-CA, 6 AL-CA, and 6 non-CA patients and were stained with a Calcium Stain Kit (ScyTek Laboratories, Logan, UT, USA). Staining was performed according to the manufacturer’s instructions; sections were immersed in silver nitrate solution (5%) for 60 min; during silver nitrate staining, the sections were irradiated with ultraviolet light at a distance of 60 cm; nuclear fast red staining was used for contrast staining of nuclei. Tumor tissue containing calcifications was used as a positive control to determine whether von Kossa staining worked (Figure S1). The presence or absence of dusty microcalcifications as reported by Thelander et al. was determined by observing one slide per sample [16].

### 4.5. Quantitative Polymerase Chain Reaction (qPCR)

Myocardial biopsy samples from 11 ATTR-CA, 6 AL-CA, and 6 non-CA patients were soaked in TRIzol reagent (Thermo Fisher Scientific, Waltham, MA, USA) and homogenized with the bead crusher µT-01 (Taitec, Koshigaya, Japan), and total RNA was extracted using the PureLink RNA Mini Kit (Thermo Fisher Scientific). Complementary DNA was prepared by reverse transcription of the extracted RNA using the SuperScript VILO Master Mix (Thermo Fisher Scientific). Primers listed in Table 4 (Integrated DNA Technologies, Coralville, IA, USA), PowerUp SYBR Green Master Mix (Thermo Fisher Scientific), and the QuantStudio 1 Real-Time PCR System (Thermo Fisher Scientific) were used for qPCR. The samples were duplicated, and the expression levels were calculated and compared using the ΔΔCT method [38]. GAPDH was used as the internal control.

### 4.6. Transmission Electron Microscopy

Tissues from two patients with ATTR-A, two patients with AL-CA, and two patients with hypertensive heart disease were studied. Endomyocardial biopsy samples were soaked in 2.5% glutaraldehyde solution, fixed at 4 °C, and subjected to the BioMedical Laboratories (Tokyo, Japan) for pathological diagnosis. Tissue blocks returned after pathological diagnosis were sent to Tokai Electron Microscopic Analysis (Nagoya, Japan) for analysis.

### 4.7. Statistics

The analyses were performed using the SPSS software program, version 24 (IBM SPSS Statistics for Windows, Version 24.0., Armonk, NY, USA). Categorical variables were compared using the chi-squared test with Bonferroni correction. The qPCR results were expressed as the mean ± standard deviation and statistically analyzed using one-way analysis of variance (ANOVA). Statistical significance was set at *p* < 0.05.

## 5. Conclusions

Similar to previous reports, more calcified microparticles were found in endomyocardial biopsy samples from patients with ATTR-CA. However, no elevated expressions of bone metabolism-related genes were observed, suggesting no involvement of active calcification-promoting mechanisms. The mechanisms explaining bone tracer accumulation in the heart of patients with ATTR-CA, which is stronger than that in the ribs, may require further investigation.

## Figures and Tables

**Table 1:** Cardiac uptake of ^99m^Tc-PYP.

Type	Perugini Grading Score			
	0	1	2	3
ATTR-CA (N = 26)	0 (0)	0/26	1 (4)	25 (96)
AL-CA (N = 6)	3 (50)	2 (33)	1 (17)	0 (0)
non-CA (N = 11)	10 (91)	0 (0)	1 (9)	0 (0)

Data are presented as number (%). AL-CA, amyloid light-chain cardiac amyloidosis; ATTR-CA, transthyretin cardiac amyloidosis; non-CA, noncardiac amyloidosis.

**Table 2:** Percentage of von Kossa-positive samples.

Type	Von Kossa-Positive
ATTR-CA (N = 13)	8 (62) ^a^
AL-CA (N = 6)	2 (33) ^a,b^
non-CA (N = 6)	0 (0) ^b^

Data are presented as number (%). Chi-square test with Bonferroni correction, *p* = 0.036. Each superscripted letter displays a subset of groups whose proportions are not significantly different from each other.

**Table 3:** Renal function and serum calcium concentration.

	ATTR-CA	AL-CA	Non-CA	*p* Value
serum creatinine (mg/dL)	1.3 ± 0.6	1.3 ± 0.6	1.5 ± 1.3	0.794
estimated glomerular filtration rate (mL/min/1.73 m^2^)	49.1 ± 17.4	48.6 ± 22.6	51.6 ± 18.4	0.930
serum calcium (mg/dL)	9.1 ± 0.4	9.3 ± 0.7	9.2 ± 0.5	0.640

One of 26 patients with ATTR-CA, one of 6 patients with AL-CA, and two of 11 patients with non-CA were on hemodialysis. Patients on hemodialysis were excluded. Data are displayed as mean ± standard deviation. ANOVA with Bonferroni test was performed.

**Table 4:** Real-time PCR primer pairs.

Gene Symbol		Sequence	Product Size (bp)
*AKNH*	F	CGATTTTGACAGCCACATACCC	100
	R	GTTGCTGGGGTTATTCTTGTCG	
*ALPL*	F	CAAAGGCTTCTTCTTGCTGGTG	70
	R	CTGCTTGGCTTTTCCTTCATGG	
*BGLAP*	F	CAGCCTTTGTGTCCAAGCAG	136
	R	TCCGGATTGAGCTCACACAC	
*BMP2*	F	GCAGCTTCCACCATGAAGAATC	144
	R	GCATCTGTTCTCGGAAAACCTG	
*BMP4*	F	ATCTTTACCGGCTTCAGTCTGG	129
	R	TCTCCAGATGTTCTTCGTGGTG	
*ENPP1*	F	CCTGTGTTGAGCTTGGAAACTG	135
	R	AGTCATCTGAACAGGCACAGAG	
*GAPDH*	F	CAACGACCACTTTGTCAAGCTC	144
	R	TCTCTTCCTCTTGTGCTCTTGC	
*RUNX2*	F	TCACAAATCCTCCCCAAGTAGC	127
	R	AGGCGGTCAGAGAACAAACTAG	
*SLC20A1*	F	GCGTGGACTTGAAAGAGGAAAC	88
	R	TGACTGAACTGGACAAGGTTCC	
*SLC20A2*	F	CGTGGATGCGGAGGAAAATAAC	75
	R	TGAGGCTTTCGTCAGATACTCG	
*SPP1*	F	ACAGACCCTTCCAAGTAAGTCC	119
	R	CATCATCAGAGTCGTTCGAGTC	
*TNFRSF11A*	F	TGGACCAACTGTACCTTCCTTG	95
	R	GGTTTTCTAGCTGGCAGAGAAG	
*TNFRSF11B*	F	GGGACCACAATGAACAACTTGC	75
	R	TTCCTGGGTGGTCCACTTAATG	
*TNFSF11*	F	ATCACAGCACATCAGAGCAGAG	78
	R	GCTTCAAGCTTGCTCCTCTTG	

F: forward primer, R: reverse primer.

## Data Availability

The data and materials supporting the results of this study can be provided by the corresponding author upon request.

## Supplementary Materials

The following supporting information can be downloaded at: https://www.mdpi.com/article/10.3390/ijms24031921/s1.

## Abbreviations

ANOVA	analysis of variance
AL-CA	amyloid light-chain cardiac amyloidosis
ATTR-CA	transthyretin cardiac amyloidosis
ATTRv	hereditary transthyretin amyloidosis
ATTRwt	wild-type transthyretin amyloidosis
H/CL	heart-to-contralateral
^99m^Tc-DPD	^99m^Tc-dicarboxypropane diphosphonate
^99m^Tc-PYP	^99m^Tc-pyrophoshate
Non-CA	noncardiac amyloidosis
TEM	transmission electron microscopy
TTR	transthyretin
qPCR	quantitative polymerase chain reaction
