# Supplementary material for: Microcalcification and 99mTc-Pyrophosphate Uptake without Increased Bone Metabolism in Cardiac Tissue from Patients with Transthyretin Cardiac Amyloidosis
Source: Int J Mol Sci. 2023 Jan 18;24(3):1921. doi: 10.3390/ijms24031921 (PMC9916282; doi:10.3390/ijms24031921)
Supplement: Supplementary file 1 [file ijms-24-01921-s001.zip › Table S1.pdf]

Supplementary Table S1. Summary of the results.

No	Sex	Age	Diagnosis	Perugini Grade Score	Microcalcification detected by von Kossa staining	Calcified particles detected by transmission electron microscope	RNA extraction	Hemodialysis	Serum creatinine (mg/dL)	Estimated glomerular filtration rate (mL/min/1.73 m^2)	Serum calcium (mg/dL)
1	M	62	AL-CA	0	-		+		1.24	46.9	10.5
2	F	57	AL-CA	0	-		+		0.57	83.1	9.0
3	M	72	ATTRwt-CA	3					1.24	44.9	9.7
4	M	76	ATTRwt-CA	3					0.87	61.6	9.2
5	M	28	non-CA	0			+	+	N/A	N/A	N/A
6	M	68	ATTRwt-CA	3					0.94	61.8	9.2
7	F	75	ATTRwt-CA	3					0.81	52.3	9.4
8	F	81	ATTRwt-CA	3					0.83	50	N/A
9	M	77	ATTRwt-CA	3					1.73	30.6	N/A
10	M	77	ATTRwt-CA	3					2.04	25.6	8.7
11	M	84	ATTRwt-CA	3					0.91	60.3	8.6
12	F	49	non-CA	0			+	+	N/A	N/A	N/A
13	M	88	ATTRwt-CA	3					0.98	54.9	8.3
14	M	82	ATTRwt-CA	3					1.33	40.1	N/A
15	M	81	non-CA	0					1.51	34.9	9.4
16	M	67	non-CA	0					1.01	57.4	9.1
17	M	77	ATTRwt-CA	3					1.01	55.2	N/A
18	M	71	ATTRwt-CA	3	+				0.82	70.9	9.3
19	M	68	ATTRwt-CA	3	+	-	+		0.88	77.3	9.5
20	M	67	non-CA	0					0.8	74.1	8.7
21	F	81	ATTRwt-CA	3	-		+		0.9	45.6	9.7
22	M	61	AL-CA	1	-	-	+		1.82	31	9.3
23	M	75	ATTRwt-CA	2	+		+		3.45	14.5	8.7
24	M	76	ATTRwt-CA	3	-		+		1.11	49.9	9.2

25	M	55	non-CA	0	-		+		0.96	64.2	9.1
26	M	77	ATTRwt-CA	3	+		+		2.4	21.4	9.3
27	M	40	non-CA	0	-	-	+		4.89	11.9	8.5
28	M	84	ATTRwt-CA	3	-		+		0.77	72.4	9.1
29	M	79	ATTRv-CA	3	+		+		1.56	34	8.9
30	M	80	non-CA	0	-				1.11	49.2	8.8
31	M	77	ATTRwt-CA	3	+		+		0.73	78.7	9.4
32	M	37	non-CA	0	-	-	+		1.11	61.4	10.2
33	F	83	ATTRwt-CA	3	-			+	N/A	N/A	N/A
34	F	77	ATTRwt-CA	3	+	+	+		1.24	32.6	9.1
35	F	74	non-CA	0	-		+		0.72	59.7	9.0
36	M	66	ATTRwt-CA	3	+		+		0.92	63.9	9.2
37	M	67	non-CA	2	-				1.11	51.8	9.7
38	M	73	ATTRwt-CA	3					1.02	55.4	9.0
39	F	63	AL-CA	2	-		+		0.86	51.5	9.3
40	M	80	AL-CA	1	+	-	+	+	N/A	N/A	N/A
41	M	84	ATTRwt-CA	3	-		+		1.44	36.5	9.0
42	M	88	ATTRwt-CA	3					1.39	37.4	8.7
43	M	76	AL-CA	0	+		+		1.99	26.4	8.8

AL-CA, amyloid light-chain cardiac amyloidosis; ATTRwt-CA, wild-type transthyretin cardiac amyloidosis; ATTRv-CA, hereditary transthyretin cardiac amyloidosis; noncardiac amyloidosis, non-CA.
