# Supplementary figures and images for: Microcalcification and 99mTc-Pyrophosphate Uptake without Increased Bone Metabolism in Cardiac Tissue from Patients with Transthyretin Cardiac Amyloidosis
Source: Int J Mol Sci. 2023 Jan 18;24(3):1921. doi: 10.3390/ijms24031921 (PMC9916282; doi:10.3390/ijms24031921)

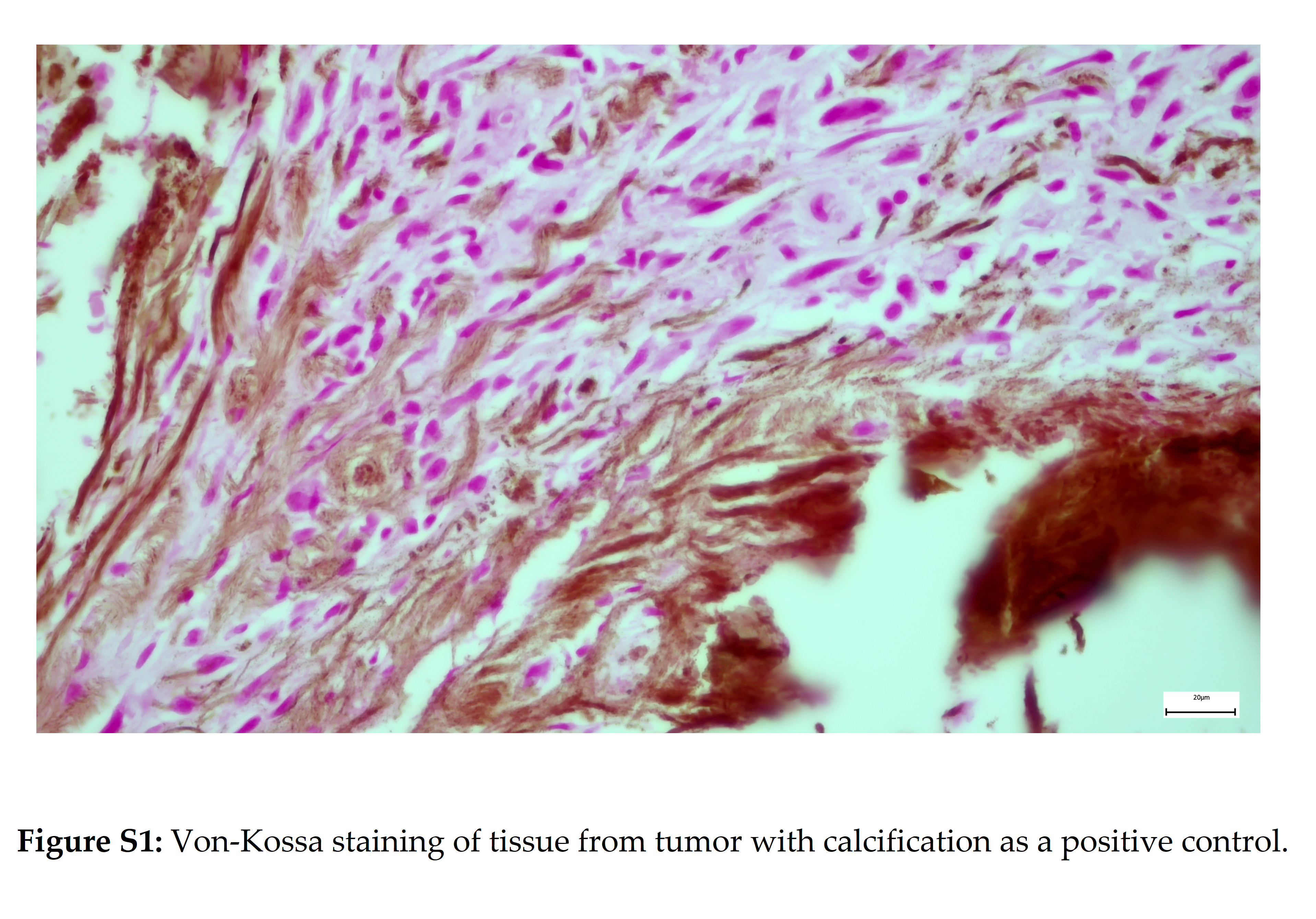

Supplement: Supplementary file 1 [file ijms-24-01921-s001.zip › Figure S1.tif]
